# Reprogramming the tumor microenvironment to boost adoptive T cell therapy

**DOI:** 10.3389/fimmu.2025.1677548

**Published:** 2025-10-23

**Authors:** Maria Fernanda Meza Pacheco, Lee-Hwa Tai

**Affiliations:** ^1^ Departement of Immunology and Cell Biology, Université de Sherbrooke, Sherbrooke, QC, Canada; ^2^ Centre de Recherche du Centre Hospitalier Universitaire de Sherbrooke, Centre Hospitalier Universitaire de Sherbrooke, Sherbrooke, QC, Canada

**Keywords:** tumor microenvironment, adoptive T cell immunotherapy, tumor infiltrating lymphocyte, CAR (chimeric antigen receptor) T-cell therapy, immunotherapy, oncology, immunomodulation

## Abstract

Adoptive T cell therapies (ACT) have revolutionized the management of hematologic malignancies; however, their efficacy in solid tumors remains limited. Accumulating evidence implicates the tumor microenvironment (TME) - a highly complex and immunosuppressive niche as a major barrier to their effectiveness. In this review, we propose that the next generation of ACT will require a fundamental shift from a reductionist focus on T cell engineering alone to an integrated approach that considers the interactions between immune cells and the TME. A comprehensive literature review identified several emerging strategies to enhance the efficacy of ACT, including reprogramming tumor vasculature, repolarizing immunosuppressive myeloid and stromal cells, leveraging oncolytic viruses to remodel antigen presentation, inducing acute sterile inflammation, and targeting the physical properties of the extracellular matrix. While many of these approaches remain in early-stage development, some have already progressed to clinical trials, indicating their potential for clinical translation. Additionally, we found that conventional therapies, such as surgery, chemotherapy, and radiotherapy, can be strategically integrated with ACT to improve therapeutic outcomes. These findings highlight a shift in the field toward more integrative approaches. Future advances will likely depend on reprogramming the TME to support T cell persistence and functions. Addressing these interconnected challenges will require closer collaboration between immunology, oncology, and bioengineering disciplines.

## 1 Introduction

Adoptive T cell therapies (ACT), including tumor-infiltrating lymphocyte (TIL) therapy and chimeric antigen receptor (CAR)-T cells, have effectively changed the treatment of hematologic malignancies (1, 2). Yet, their impact on solid tumors has remained disappointingly limited (3). This gap underscores a critical paradox: despite advances in engineering, ever more potent immune effector cells, durable responses in solid tumors remain rare, highlighting a pressing need to better understand the biological and microenvironmental barriers that underlie this resistance.

A growing body of evidence points not to the T cells themselves, but to the complex and suppressive ecosystem in which they must operate, the tumor microenvironment (TME) (4). While ACT strategies have traditionally focused on enhancing intrinsic T cell potency and antigen specificity, they fail to address the extrinsic barriers imposed by the TME, including immune exclusion, metabolic suppression, stromal sequestration, and dysfunctional antigen presentation (5).

In this review, we propose that the next generation of ACT will require a fundamental shift, from a reductionist focus on T cell enhancement to an integrated approach that considers the reciprocal and dynamic interactions between immune cells and the TME. By harnessing multidisciplinary insights into stromal biology, tissue immunology, and tumor metabolism, we can begin to reshape the TME into an environment that supports rather than suppresses ACT. We will highlight emerging strategies that synergize ACT with interventions targeting the TME, from *in situ* antigen presenting platforms and myeloid re-education to stromal remodeling and vascular normalization. Together, these approaches offer a blueprint for unlocking the full potential of ACT in solid tumors.

## 2 Tumor microenvironment: complexity and barriers

If ACT has proven transformative in hematologic cancers, why does it fail in solid tumors? Despite both tumor types sharing fundamental malignant traits, as outlined in the widely accepted “hallmarks of cancer” (6). The answer lies, at least in part, in the uniquely complex, immune responsive and immunosuppressive nature of the TME. Unlike the relatively accessible architecture of blood malignancies, solid tumors are entrenched within a fortified stromal and cellular network that actively resists immune-mediated destruction (7).

Broadly, the TME can be grouped according to the extent of immune cell infiltration. As Wu et al., explain tumors are generally divided into immune-inflamed (“hot”), immune-excluded (“altered”), or immune-desert (“cold”) phenotypes (8). Hot tumors are characterized by abundant T cell infiltration, high PD-L1 expression, and elevated tumor mutational burden, all of which are features associated with improved responsiveness to immune checkpoint inhibitors (ICIs). In contrast, immune-excluded tumors restrict CD8^+^ T cells to the surrounding stroma, preventing their entry into the tumor core, while immune-desert tumors show a near-complete absence of CD8^+^ T cells both within and around the tumor (8). These non-inflamed tumors frequently accumulate immunosuppressive populations, including tumor-associated macrophages (TAMs), regulatory T cells (Tregs), and myeloid-derived suppressor cells (MDSCs). As a result, altered and cold tumors are marked by weak endogenous antitumor immunity and limited clinical benefit from ICI therapy (8).

**Figure 1:**
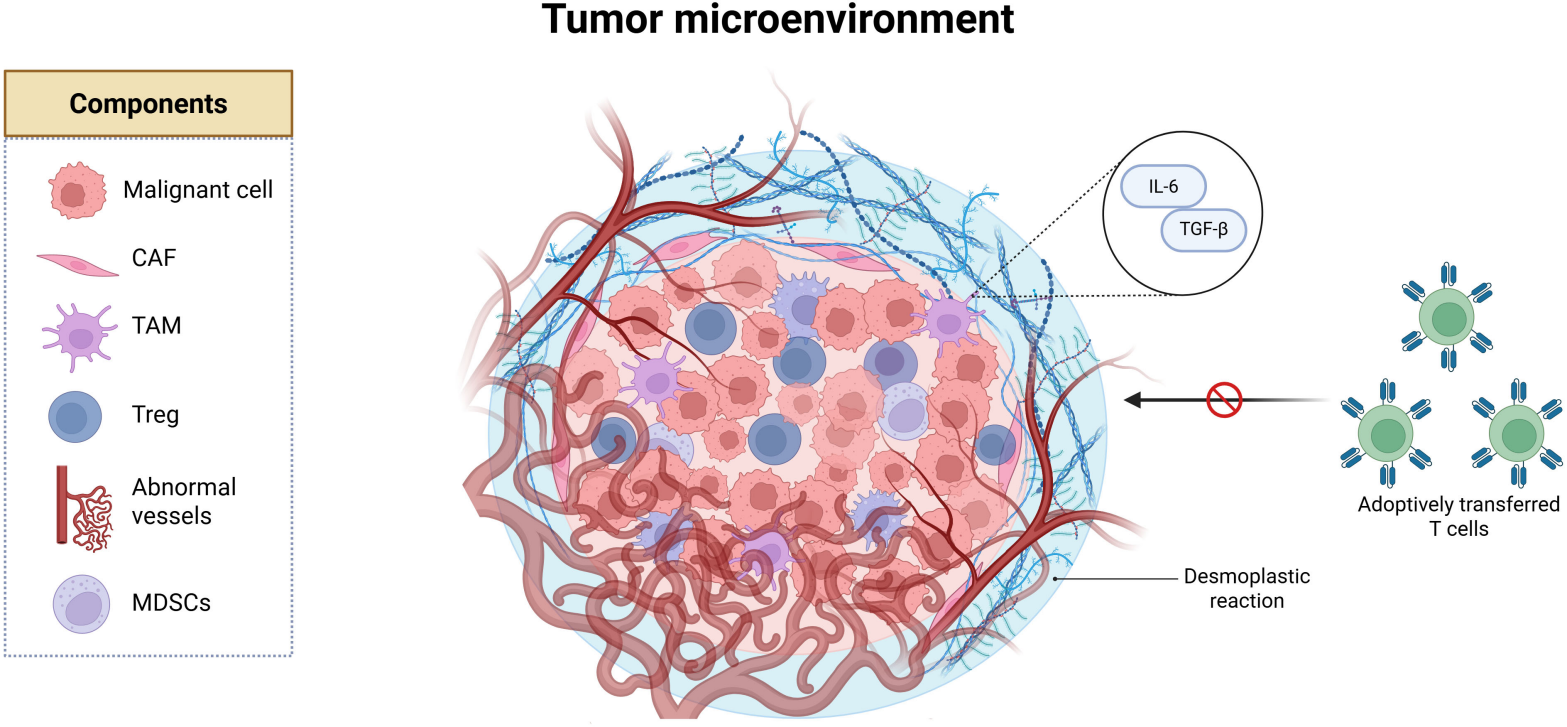
The tumor microenvironment (TME) is composed of malignant cells, cancer-associated fibroblasts (CAFs) that remodel the extracellular matrix and drive a desmoplastic reaction characterized by fibrotic deposition, which acts as a physical barrier to immune infiltration. The TME also includes tumor-associated macrophages (TAMs), regulatory T cells (Tregs), and myeloid-derived suppressor cells (MDSCs), which secrete immunosuppressive cytokines, as well as endothelial cells that form abnormal blood vessels. Created in BioRender. Tai, L (2025).

Besides the infiltrating immune population or its absence, other non-malignant cell components of the TME function as active participants in tumor immune evasion. Malignant cells are surrounded by endothelial cells, pericytes, and resident tissue cells like adipocytes and neurons. These are embedded in a remodeled extracellular matrix (ECM), forming a dynamic ecosystem that fosters tumor progression while subverting immune responses (8). CAFs, for instance, orchestrate ECM deposition creating a fibrotic and dense physical barrier known as “desmoplastic reaction” that limits immune infiltration. Meanwhile, abnormal vasculature contributes to hypoxia, and physical exclusion of ACT (**Figure 1**) (9).

Importantly, the TME is not uniform across cancer but is profoundly shaped by the tumor’s tissue of origin. Brain tumors such as glioblastoma, for instance, develop within an immune-privileged organ where the blood–brain barrier restricts immune trafficking and resident microglia often adopt immunosuppressive phenotypes, creating a TME that is particularly hostile to ACT (10, 11). By contrast, desmoplastic tumors such as pancreatic ductal adenocarcinoma are dominated by cancer-associated fibroblasts and dense extracellular matrix, which act as physical and biochemical barriers to T cell infiltration (12, 13). Other tumor types, such as breast cancers, feature strong hormonal and stromal influences that modulate immune cell composition and checkpoint expression (14). Even within the same immune classification (hot, altered, or cold), these tissue-specific factors dictate which immunosuppressive mechanisms prevail, whether it be microglia and astrocytes in the brain, stellate cells in the pancreas, or adipocytes in the breast. Recognizing how the cell of origin and tissue context sculpt the TME is critical for tailoring ACT strategies, since interventions that succeed in one tumor type may fail in another due to fundamentally different microenvironmental constraints.

Building on these concepts, we first examine how conventional cancer therapies, originally developed without immunological intent, can exert significant immunomodulatory effects. Increasing evidence (15) suggests that these therapies, including chemotherapy, radiotherapy, and targeted agents, can reprogram the TME, either promoting or hindering immune responses. By understanding and leveraging their immunogenic potential, we highlight opportunities to rationally combine them with ACT to shift the TME from an immunosuppressive to an immunostimulatory state.

We then explore emerging therapeutic strategies that are explicitly designed to remodel the TME, addressing both its immunological and structural barriers. These novel approaches provide innovative avenues to enhance the infiltration, persistence, and function of ACT, and represent a critical step toward improving ACT efficacy across solid tumor contexts.

## 3 Conventional therapies and their impact on tumor immunogenicity and ACT

For decades, conventional treatments like chemotherapy, radiotherapy, and surgery have served as the backbone of cancer therapy, cutting down tumors with the blunt force of toxicity or the precision of a scalpel. Beyond their cytotoxic reputation, these treatments also exert immunomodulatory effects; they can also awaken the immune system (15). Their immunomodulatory effects, once considered collateral or negligible, are now recognized as key players in sculpting lasting anti-tumor immunity.

Both chemotherapy and radiotherapy can trigger a particular kind of cell death known as immunogenic cell death (ICD), a molecular “fire alarm” that calls the immune system into action (16). As tumor cells die, they release damage-associated molecular patterns (DAMPs) like calreticulin, HMGB1, ATP, and heat-shock proteins (15, 16). These molecules act like red flags, signaling danger to dendritic cells, encouraging them to mature and present tumor antigens, effectively turning the dying tumor into an *in situ* vaccine (15, 16). For example, chemotherapy agents like anthracyclines and oxaliplatin are well-known instigators of ICD, capable of cracking open tumors and releasing antigens that help rally CD8^+^ T cell responses (15).

However, the immune effects of chemotherapy are often context-dependent, and not always beneficial. For instance, the standard lymphodepleting regimen of cyclophosphamide (Cy) plus fludarabine (Flu), regularly administered before ACT transfer, has been linked to increased accumulation of myeloid-derived suppressor cells (MDSCs) in both preclinical models and patient samples. *In vivo* studies show that lymphodepletion triggered an IL 6–driven expansion of MDSCs, which suppressed ACT and limited their anti-tumor efficacy (17). Similarly, cyclophosphamide has been shown to skew myeloid differentiation toward immunosuppressive MDSC populations, further dampening T cell function. However, conflicting evidence suggests that a more intense Cy/Flu regimens correlates with increased serum levels of cytokines like MCP-1 and IL-7, which can enhance T cell persistence and improve progression-free survival (18). These findings highlight the nuance of these therapeutic strategies, considering that lymphodepletion may improve ACT outcomes, it can simultaneously promote immunosuppressive myeloid expansion that can undermine therapeutic benefit.

Surgery, the oldest and often most definitive cancer therapy, is no exception. While it can debulk or even eliminate tumors, it also risks triggering a postoperative immunosuppressive state that could lead to recurrence or metastasis. Stress hormones like cortisol peak after surgery have been linked to reduced immune surveillance (19). In patients undergoing laparoscopic resection for colorectal cancer, a transient dip in immune activity was observed, characterized by reduced T cell and NK cell function, weakened monocyte antigen presentation, and a tilt toward immunosuppressive phenotypes that lasted up to a week after surgery (19). According to Zhou et al., surgery induces a transient inflammation state caused by tissue trauma. This is followed by a period of immunosuppression; whose physiological role is to dampen excessive inflammation and prevent tissue necrosis. However, this temporary immunosuppressive state can be exploited by cancer cells to escape immunosurveillance (19).

A landmark preclinical study by Tai et al. places natural killer (NK) cells at the center of this phenomenon (20). In their preclinical models, surgically stressed animals lacking functional NK cells developed twice as many lung metastases as their non-surgical counterparts. To rule out contributions from other immune cells, the team conducted adoptive transfers of NK cells from both stressed and non-stressed donors. Only NK cells from unstressed mice conferred protection, confirming that surgery impairs NK function in a way that directly promotes metastatic outgrowth. This effect held true regardless of mouse strain, cell type, anesthesia, or surgical method, cementing the central role of NK cells in the postoperative window (20). To test translational relevance, they sampled blood immune cells from cancer patients before and after surgery. NK cell cytotoxicity dropped sharply after surgery but returned to baseline by day 28. In two patients treated with neoadjuvant JX-594-GFP^+^/βgal^-^, an oncolytic vaccinia virus, NK activity increased post-treatment, suggesting that perioperative oncolytic viruses (OV) might help restore anti-tumor immunity even in humans. Though the patient cohort was small, the findings offer a compelling case for combining surgery with immune stimulation to prevent postoperative relapses (20).

Radiotherapy, on the other hand, has been shown to reprogram the TME to become more receptive to immunotherapy (21, 22). Preclinical studies show that irradiating tumors before ACT can prime the TME, activating dendritic cells, improving antigen uptake, and promoting better cross-priming of T cells. Radiation also boosts T cell infiltration, likely by increasing expression of chemo-attractants like CCL5, CXCL9, and CXCL11. T cells harvested from irradiated tumors show higher levels of effector cytokines like IFN-γ and TNF-α, suggesting that radiation doesn’t just invite T cells into the tumor, but also helps them fight it more effectively (21, 22). These immunomodulatory effects of radiotherapy suggest that strategic integration of irradiation with ACT, particularly TIL therapy may boost the quality, functionality, and trafficking of infused T cells by conditioning the TME to be more permissive and immunologically active.

While chemotherapy, radiation, and surgery are mainstays of cancer therapy, the field has steadily shifted toward precision therapies, interventions finely tuned to target molecular drivers unique to each tumor. These include small molecule inhibitors (23), hormone-targeting agents (24), and first-generation immunotherapies, often guided by biomarkers like EGFR or HER2 (25). But their integration into ACT is limited by patient-specific factors. By contrast, conventional treatments are still broad-spectrum tools, widely used and widely applicable. Despite being designed with tumor-killing, not immune-modulating goals, they hold surprising potential as partners for ACT. This review focuses on these versatile, frontline therapies, which may serve not just as blunt instruments, but as strategic allies in reprogramming the TME for immune success.

## 4 Novel experimental therapies targeting the TME

**Figure 2:**
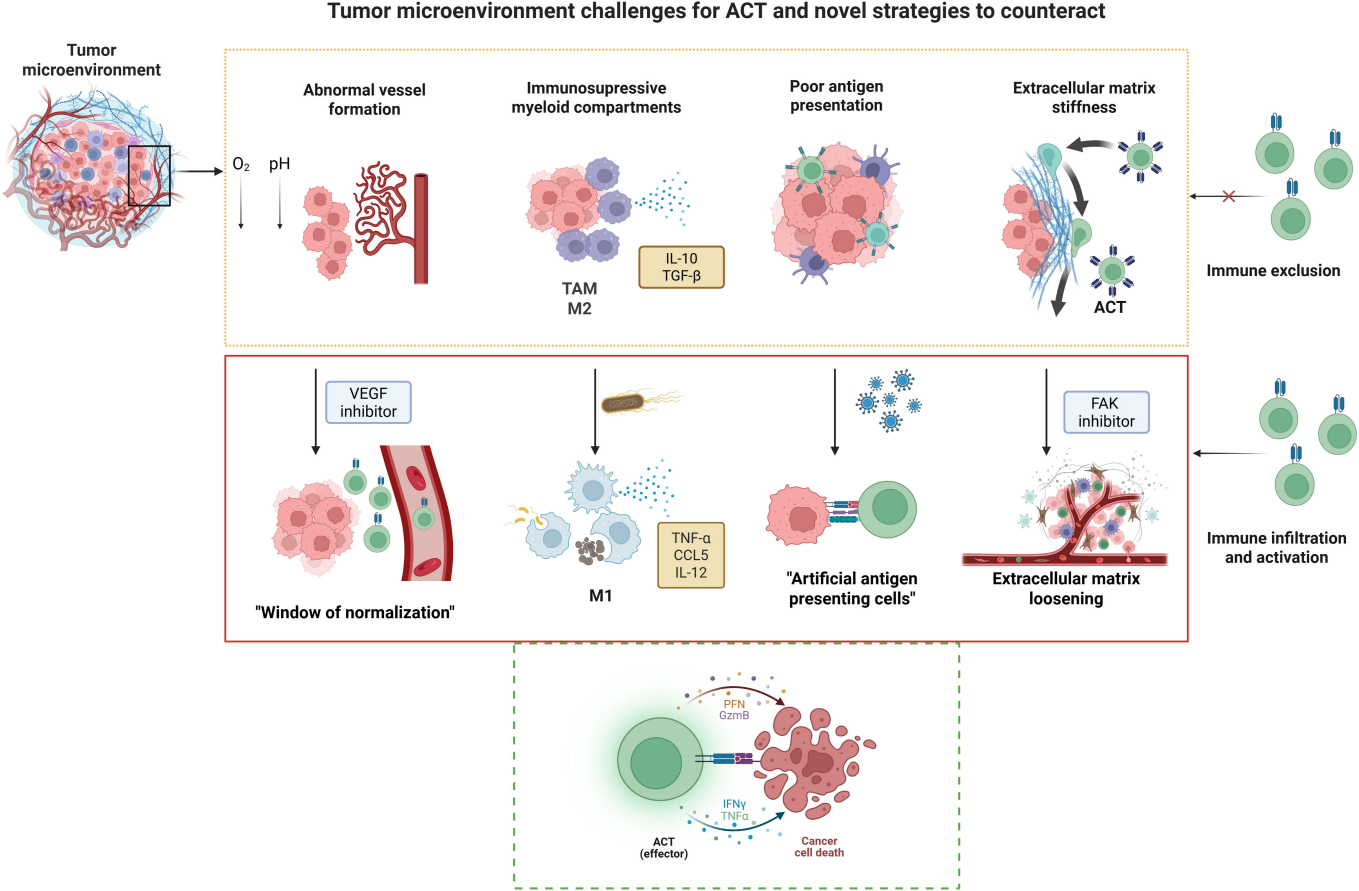
Tumor microenvironment challenges for ACT and novel strategies to counteract. Solid tumors pose unique challenges for adoptive cell therapy (ACT), including abnormal vasculature, immunosuppressive myeloid populations such as tumor-associated macrophages (TAMs) and alternatively activated macrophages (M2), poor antigen presentation, and a stiff extracellular matrix (ECM). These barriers can be addressed through innovative strategies such as vascular endothelial growth factor (VEGF) inhibition to normalize blood vessels, engineered bacteria to stimulate cells from the innate immunity including classically activated macrophages (M1); oncolytic viruses (OVs) to convert tumor cells into artificial antigen-presenting cells, and focal adhesion kinase (FAK) inhibition to remodel the ECM and enhance immune infiltration. Together, these approaches promote effector T cell activation and facilitate tumor cell destruction. Created in BioRender. Tai, L (2025).

As emphasized throughout this review, the TME is not a passive backdrop, but a dynamic, multifaceted ecosystem composed of immune and stromal cells, vasculature, extracellular matrix, and soluble mediators that profoundly shape the outcome of anti-tumor immune responses. Rather than viewing the TME as a barrier to overcome, emerging therapeutic strategies are increasingly focused on actively reprogramming it to support immune-mediated tumor clearance (**Figure 2**). In the following section, we highlight experimental approaches that target key aspects of the TME, including vascular remodeling, immune cell re-education, extracellular matrix modulation, induction of localized inflammation, and enhancement of antigen presentation. These strategies not only hold promise as standalone interventions but also as powerful adjuvants to ACT, with the potential to enhance T cell infiltration, persistence, and effector function within otherwise hostile tumor niches.

### 4.1 Reprogramming tumor vasculature

A defining hallmark of cancer is its uncontrolled proliferation, characterized by cells that evade normal growth restraints and exploit host resources to sustain their expansion. However, this unchecked growth rapidly surpasses the capacity of the local blood supply, resulting in regions of hypoxia and nutrient deprivation (26). To overcome these limitations, tumors initiate angiogenesis, a process by which new blood vessels are generated to meet the heightened metabolic demands of the malignant tissue (26).

Yet, tumor-induced angiogenesis rarely produces clean, efficient vessels. Instead, the resulting vasculature is chaotic and dysfunctional, leaky, poorly connected, and unevenly branched (27). As described by Stylianopoulos et al., this disarray contributes to key features of the TME, including hypoxia, acidosis, and immunosuppression (27). These vascular defects are more than just structural, they’re immunological roadblocks. Proper immune surveillance requires a well-perfused, accessible network, but tumor vasculature often denies immune cells entry (28). For ACT, this poses a major challenge, considering that even the best-engineered T cells can’t act if they can’t reach their targets. Poor perfusion limits trafficking, dampens antigen presentation, and restricts therapeutic delivery deep into the tumor core (28).

Several culprits contribute to this dysfunction. For instance, leaky vessels flood the interstitial space, narrow and winding vessels that resist normal flow, and even collapsed vessels, compressed by swelling tumors and fibrotic pressure (28). However, this chaotic vasculature is reversible. A clinical trial using an oral tyrosine kinase inhibitor targeting VEGF receptors showed that vessel normalization is possible. By reducing hyperpermeability and leakage, this approach alleviated intracranial edema in glioblastoma patients who had exhausted standard treatments (29). Anti-VEGF therapy also boosted the impact of chemotherapy and radiotherapy, but only during a critical “window of vascular normalization” (30). Prolonged treatment leads to toxicity, and once the drug is withdrawn, the vasculature reverts to its dysfunctional state. These setbacks reveal both the promise and limitations of vascular normalization, highlighting the need for safer, more durable strategies (30). Still, this transient window may offer a unique opportunity to synergize with ACT, creating a brief but crucial opening for ACT to navigate the vasculature and reach their tumor targets.

### 4.2 Remodeling myeloid compartments with bacterial antigens

Another major roadblock in the TME is formed not by vessels, but by TAMs, particularly those skewed toward an M2-like, immunosuppressive phenotype. These TAMs act like security guards working *for* the tumor (31), their presence impairs cytotoxic T lymphocytes (CTLs), reducing infiltration, persistence, and killing capacity, and ultimately blunting the effects of ACT. Reprogramming TAMs toward a pro-inflammatory, M1-like state has emerged as a critical strategy to break through this barrier (31).

These TAMs impair CTL functions through several mechanisms, including the expression of checkpoint ligands such as PD-L1 and the secretion of immunosuppressive cytokines like IL-10 and TGF-β (32). They also contribute to immune exclusion by remodeling the ECM and creating chemokine gradients that retain T cells in the stromal regions, preventing their infiltration into tumor nests (32). Notably, Peranzoni et al. demonstrated that TAMs can establish a T cell–excluded tumor phenotype, thereby severely limiting the capacity of CD8^+^ T cells to access and eliminate tumor cells (32). Collectively, these effects create a hostile immune landscape that diminishes T cell persistence, trafficking, and cytotoxic activity, ultimately reducing the therapeutic benefit of ACT (32). Therefore, strategies that deplete or reprogram TAMs toward a pro-inflammatory, M1-like phenotype are increasingly recognized as essential to overcoming these barriers

Addressing this problem, recent work by Zhu et al., used non-pathogenic *Escherichia coli* MG1655 (33). In mouse models of melanoma, intratumoral injection of this bacterial strain led to selective colonization of hypoxic tumor regions, exactly where immune suppression tends to thrive. There, the bacteria acted as a biological switch, triggering TAM reprogramming through TLR4 activation by bacterial surface components like LPS and flagellin (33). This microbial wake-up call activated NF-κB signaling, pushing macrophages toward an M1-like identity characterized by increased IL-12, TNF-α, MHC-II, and CD86 expression. These reprogrammed macrophages then secreted CCL5, establishing a chemokine gradient that pulled CD8^+^ T cells into the heart of the tumor (33). To test how this bacterial intervention worked alongside ACT, the authors used the B16F10-OVA melanoma model, transferring OT-I CD8^+^ T cells specific to ovalbumin. The results were striking tumors treated with both MG1655 and T cells showed enhanced infiltration, elevated effector cytokines like IFN-γ and TNF-α, greater tumor regression, and most importantly longer survival. The success came from both TAM reprogramming and vascular normalization, together reshaping the TME into one that supports, rather than suppresses, immune function (33).

These findings suggest that microbial-based therapies can serve as local immune boosters, rewiring the TME to make ACT more effective. Using safe, non-pathogenic bacteria like *E. coli* MG1655 could offer a targeted, low-toxicity option to jumpstart immune responses where they’re needed most. Of course, many questions remain, especially regarding translation to humans, where tumors lack engineered antigens and where bacterial colonization may be harder to achieve. But this work points to an exciting frontier, that of leveraging the body’s innate microbial sensing systems to tip the balance in favor of anti-tumor immunity.

### 4.3 Oncolytic viruses as *in situ* antigen-presenting platforms

One of the key barriers to successful ACT in solid tumors isn’t just getting T cells into the tumor, it’s keeping them switched on once they arrive. Many tumors create a barren immunological landscape that includes poor antigen presentation, MHC downregulation, missing co-stimulatory signals, and a tolerogenic TME that all contribute to leave T cells under-stimulated and ineffective (34). This results in the failure of TIL to maintain their cytotoxic activity, leading to therapeutic failure.

To overcome this, newer strategies aim not only to enhance T cell infiltration but to reprogram the tumor itself into a hub of immune activation. One particularly creative approach uses OVs, viruses that selectively infect and lysis cancer cells, which are engineered to transform those same tumor cells into “artificial antigen-presenting cells” (aAPCs) (34). These OVs exploit the tumor’s natural vulnerability to viral infection and turn it into a Trojan horse, delivering immunostimulatory molecules right to the tumor core.

Ye et al. developed a vaccinia virus armed with OX40L and interleukin-12 (IL-12) to force infected tumor cells into becoming immune-activating platforms (34). Once inside, the virus didn’t just kill the tumor, it rewired the surviving cells to express co-stimulatory molecules and inflammatory cytokines. These newly “converted” tumor cells were able to engage TILs, enhancing their activation, sparking epitope spreading, and reducing tumor burden. When combined with TILs, this strategy yielded particularly strong responses. It also reshaped the surrounding TME, including repolarization of immunosuppressive macrophages toward an M1-like, pro-inflammatory state (34). What sets this approach apart is its ability to relocate the site of antigen presentation. Rather than relying on dendritic cells (DCs), which are often scarce or dysfunctional in tumors. This method turns the tumor cells themselves into pseudo-APCs, handing the immune system a clear signal on what to attack and the co-stimulatory signals they require for their functions. This contrasts with DC-based vaccines, which require ex vivo priming of dendritic cells with tumor antigens before reinfusing them into the patient (35). While strategies like Sipuleucel-T, the first FDA-approved DC vaccine for metastatic prostate cancer, have demonstrated proof of concept, their impact in solid tumors has been modest, largely due to poor DC migration and limited *in situ* antigen presentation (36).

Oncolytic viruses, by contrast, bring dual firepower. First, they induce direct lysis of infected tumor cells, spilling out a broad array of tumor-associated antigens (TAAs), some of which may not have been targeted by the original T cell population (37). Second, they deliver pathogen-associated molecular patterns (PAMPs) and DAMPs, which are molecular signatures of infection that trigger innate sensing pathways like TLRs and STING, sparking a wave of type I interferon signaling and chemokine release (37). This brings both innate and adaptive immune players into the fight, creating a coordinated immune assault.

Several clinical candidates are already building on this logic. For example, Talimogene laherparepvec (T-VEC), a herpes simplex virus engineered to express GM-CSF, is FDA-approved for advanced, unresectable melanoma and represents the first generation of OV therapies (38). But newer designs, like Ye et al.’s aAPC-converting virus may unlock even greater synergy with ACT, particularly in immunologically “cold” tumors, where T cells often remain inactive despite being present (34).

Yet not all data points toward synergy. Some studies complicate the narrative, revealing a more nuanced and sometimes counterintuitive relationship between OV and ACT. In a striking example, Evgin et al. used a B16 melanoma model expressing EGFRvIII, a mutant form of the epidermal growth factor receptor, to evaluate the interplay between murine EGFRvIII-targeted CAR-T cells and VSVmIFNβ, a recombinant vesicular stomatitis virus engineered to express murine interferon-beta (39). The hypothesis was that the virus would induce immunogenic tumor cell death and chemokine release, while the CAR-T cells would specifically target and kill EGFRvIII-expressing tumor cells, leading to enhanced tumor control through synergistic effects (39). Instead, the researchers observed the opposite effect. While OV infection did stimulate chemokine production (including IFN-α, CXCL10, and CCL5), which should have supported T cell recruitment, they found a significant loss of CAR-T cells following viral administration. This attrition coincided with peak levels of intratumoral IFN-β, pointing to a deleterious effect of type I interferons on CAR-T cell persistence. The authors concluded that, contrary to expectations, IFN-β may impair CAR-T cell expansion within the tumor. They proposed several strategies to overcome this obstacle, including temporal separation of the two therapies or engineering CAR-T cells resistant to IFN-β signaling (39). To empirically test the temporal spacing hypothesis, the researchers delivered the first dose of OV in a lymphodepleted *in vivo* model 5 days after the administration of CAR-T cells, which led to a decrease in IFN-β levels intratumorally. This in combination with checkpoint inhibitors to counteract the residual IFN-induced effects of the CAR-T cells may allow proper synergistic and mutually potentiating effects for ACT and OVs.

Altogether, these strategies signal a paradigm shift. Rather than bolstering antigen presentation through external dendritic cells or adjuvants, why not reprogram the tumor itself into a beacon for immune activation? OVs offer this possibility (37). For ACT, this could be a game-changer, allowing ACT to not only find their targets, but to be reactivated, sustained, and empowered right at the tumor site, supporting long-term control and immune memory. Still, as studies like Evgin et al. remind us, the promise of synergy comes with caveats (39). Type I interferons, while beneficial for innate immune activation, may compromise the persistence of therapeutic T cells. Understanding and refining these dynamics such as timing, dosing, and genetic payloads, will be crucial. The path forward lies not only in arming T cells or viruses more aggressively, but in choreographing their interplay with preci**sion.**

### 4.4 Acute inflammation via cryo-thermal therapy

Besides microbial-based strategies that introduce foreign antigens to stir up immune activity, another promising avenue to enhance ACT lies in harnessing “sterile” inflammation, which is an immune response ignited without any infection (40). The idea is to flip the immunological switch in cold tumors, turning them “hot” by unleashing danger signals, mobilizing immune cells, and fostering a supportive environment for both endogenous and ACT (41).

To accomplish this, Wang et al. developed a cryo-thermal therapy (CTT) platform that cleverly combines two extremes, freezing and heating in a single cycle. Liquid nitrogen cryoablation is followed by localized radiofrequency-induced hyperthermia, triggering the release of DAMPs and sparking a localized but powerful wave of sterile inflammation (42). Previous studies from the same group showed that this approach doesn’t just ablate tumors, it transforms the immune landscape. In melanoma-bearing mice, CTT reduced metastasis, extended survival, and reshaped systemic immunity. MDSCs decreased, while activated CD4^+^ and CD8^+^ T cells increased across blood, spleen, and lungs. Macrophages, too, shifted gears, moving from an immunosuppressive (M2) to a pro-inflammatory (M1) phenotype (40–42).

But could this inflammatory spark fuel a stronger ACT response? To test this, the authors combined CTT with ACT in mouse models of melanoma (B16F10) and advanced breast cancer (4T1) (42). CD3^+^ T cells were harvested from tumor-bearing mice, expanded ex vivo, and reinfused after thermal treatment. Tumors were established bilaterally, the right tumor received treatment, meaning either CTT and ACT in combination, CTT alone or ACT alone, while the left tumor was left untreated; acting as a control to test whether the immune response could spread systemically. The results were remarkable, in the combination group, even the untreated tumors showed signs of immune activation, upregulated gene signatures tied to neutrophil chemotaxis, monocyte recruitment, dendritic cell priming, and M1 macrophage polarization. Histology confirmed this immune domino effect with the evidence of necrosis in distant tumors only when CTT and ACT were combined. Individually, neither therapy could stop the progression of distant tumors. But together, they worked in concert to suppress tumor growth systemically. In the metastatic 4T1 model, this synergy translated to a dramatic improvement in survival, from roughly 50% to nearly 90% in the long term (42).

This approach reframes the way we think about T cell-based therapies. Rather than relying on pathogen-associated cues, cryo-thermal therapy induces immune awakening through tissue stress itself, making the tumor its own alarm bell. By priming the environment for T cell entry, activation, and persistence, CTT may be what ACT needs, an inflammation without infection, but with impact.

### 4.5 ECM stiffness and immune exclusion

Like the walls of a fortress, the ECM in solid tumors doesn’t just hold tissue together, it can keep immune cells out. More than just structural scaffolding, the ECM serves as a dynamic regulator of the TME, shaping how cells behave, migrate, and survive. In many desmoplastic tumors, such as pancreatic ductal adenocarcinoma (43), this matrix becomes excessively stiff and fibrotic, largely due to hyperactive cancer-associated fibroblast (CAFs) laying down thick layers of collagen, fibronectin, and other proteins.

This physical armor does more than impair blood flow and drug penetration. It also builds an immunological barrier (44, 45). T cells, especially ACT, struggle to breach these dense networks. And even if they do, they may find themselves in hostile territory. The increased stiffness of the ECM activates mechanosensitive pathways like YAP/TAZ, which support tumor cell survival while skewing immune and stromal cell gene expression toward immune suppression (46).

In a recent study, Jahin et al. peeled back the layers of this process, revealing how ECM stiffness fuels focal adhesion kinase (FAK) and integrin β1 signaling, which together activate YAP/TAZ. This pathway not only stiffens the tumor’s architecture but also dampens immune responses. When researchers inhibited FAK or integrin β1 in mouse models, the matrix loosened, immune cell infiltration improved, and genes linked to immune evasion were downregulated (46). Another study by Chitty et al. zeroed in on lysyl oxidase (LOX) enzymes, key collagen crosslinkers, as upstream enforcers of matrix rigidity, suggesting that impairing these enzymes could soften the ECM and restore immune access (47).

Still, this isn’t a simple “break down the wall” story. The ECM also helps maintain tissue organization and prevents metastasis (48). Tear it down too far, and the consequences could be just as dangerous. The challenge, then, is not destruction, but softening the matrix just enough to relieve pressure and allow ACT to infiltrate the tumors, without compromising tissue integrity (49). Encouragingly, clinical efforts are already under way. In a recent trial, Wang-Gillam et al. tested the FAK inhibitor defactinib alongside checkpoint inhibitors and chemotherapy in patients with desmoplastic tumors (50). Biopsies post-treatment revealed increased infiltration of cytotoxic and effector T cells, echoing the immune reactivation seen in preclinical models (47). The strategy didn’t just make theoretical sense; it showed early clinical promise (50).

In essence, the fibrotic ECM is more than a passive structure, it’s a key player in tumor immune resistance. Rewiring ECM-related signaling pathways could transform the TME from a barrier into a bridge, enabling ACT and other immunotherapies to reach their full potential in tumors once considered impenetrable (51).

### 4.6 Cytokine modulation to sensitize the TME for ACT

Finally, an essential aspect of the TME that must be considered to enhance ACT efficacy is the signals it provides, not only to tumor cells, but also to surrounding non-malignant components. These signals can be either pro-tumorigenic or anti-tumorigenic, thereby influencing whether ACT responses are sustained or impaired. Among these are cytokines, small, secreted proteins that mediate cell communication, and that play a particularly critical role by regulating immune activation, suppression, and survival. Cytokines profoundly shape the outcome of anticancer immunotherapies and, most notably for this review, the success or failure of ACT (52).

In a comprehensive review on the subject, Yi et al. (53) outlined multiple strategies through which cytokines can exert therapeutic, anti-tumor effects within the TME and synergize with ACT. One approach discussed previously in this review is the reprogramming of tumor vasculature, where blockade of VEGF receptors can normalize blood vessel formation and facilitate T-cell trafficking into the TME. Building on this, we now turn to a more specific focus: how cytokine modulation influences the persistence and long-term functionality of the transferred T cells, a key determinant of durable ACT efficacy.

Among the cytokines most relevant to ACT, interleukin-2 (IL-2), IL-7, and IL-15 stand out as key regulators of T-cell persistence and function (53). IL-2 has long been administered as an adjuvant in ACT protocols to promote the proliferation, activation, and survival of infused T cells (54). While high-dose IL-2 can enhance antitumor activity, it is also associated with significant toxicity, limiting its clinical applicability. Notably, Ellebaek et al. investigated a regimen combining ACT with autologous TILs and low-dose IL-2 in patients with metastatic malignant melanoma. Despite the small cohort size (n=6), this approach yielded encouraging outcomes, including reduced toxicity and durable complete responses in some patients. These findings underscore the potential of low-dose cytokine support as a strategy to improve the safety and therapeutic efficacy of ACT, warranting further exploration in larger clinical trials (54).

In parallel, Interferon-γ (IFN-γ) plays a complementary role by reshaping the TME to support ACT. Beyond its direct cytotoxic functions, IFN-γ enhances tumor immunogenicity through upregulation of MHC molecules, facilitates T-cell trafficking via induction of chemokines such as CXCL10, and can act on stromal and endothelial cells to remodel tumor vasculature in ways that favor immune infiltration (55). As an example, Larson et al. demonstrated that CAR-T cell cytotoxicity in solid tumors critically depends on tumor-intrinsic IFNγ receptor (IFNγR) signaling, a requirement not observed in hematologic malignancies (55). Using CRISPR screens and *in vivo* models, the study showed that IFNγ produced by CAR-T cells engages IFNγR on tumor cells, leading to upregulation of adhesion molecules such as ICAM-1, which stabilize immunologic synapses and enable effective killing. Loss of IFNγR signaling impaired CAR-T tumor cell binding, reduced cytotoxic efficiency, and conferred resistance to therapy, while restoring ICAM-1 expression rescued susceptibility. Importantly, this dependency was consistent across multiple solid tumor types, underscoring a fundamental difference between solid and hematological tumors in their interaction with CAR-T cells. These findings highlight tumor-intrinsic IFNγR signaling as a key determinant of CAR-T efficacy in solid cancers and suggest that strategies to enhance adhesion or IFNγR pathway activity may improve therapeutic outcomes (55).

Together, these mechanisms illustrate how targeted cytokine support can be leveraged to improve ACT durability and therapeutic outcomes, emphasizing the importance of integrating cytokine modulation strategies into adoptive immunotherapy design.

## 5 Conclusion

ACT holds immense promise for cancer treatment, yet its success in solid tumors depends on more than just T cell potency, it hinges on the hostile terrain those cells must navigate. Conventional therapies such as chemotherapy and radiotherapy can prime tumors by enhancing antigen release and facilitating immune cell infiltration, but they rarely overcome the structural and immunosuppressive barriers of TME. Still, the often-overlooked immunomodulatory effects of these treatments merit renewed attention. Could their strategic use in neoadjuvant or adjuvant settings enhance ACT efficacy? Even if ACT alone proves insufficient, it might lower the need for high doses of poorly tolerated therapies like chemotherapy. Just as the integration of surgery, chemotherapy, and radiotherapy has improved patient outcomes in the past, immunotherapy, and specifically ACT, represents the next pivotal addition to the therapeutic arsenal.

Working to achieve this goal, a growing number of strategies seek not only to empower T cells, but to recondition the environment they face. As reviewed here (**Table 1**), vascular normalization can transiently improve immune access. Cryo-thermal therapy induces sterile inflammation, recruiting innate effectors that synergize with ACT. Targeting the fibrotic stroma, via inhibition of FAK, or LOX enzymes, softens the extracellular matrix and reverses T cell exclusion. OVs offer another route by turning tumor cells into antigen-presenting platforms. As a key example of the translational potential of these concepts, an ongoing Phase I clinical trial is recruiting patients with HER2-positive cancers to evaluate a combination of autologous HER2 CAR-T cells and intratumoral administration of CAdVEC, an oncolytic adenovirus. This approach aims to enhance the therapeutic efficacy of both modalities and highlights that TME remodeling to support ACT is not merely hypothetical but an active strategy under clinical investigation (56).This reflects a necessary shift from enhancing immune effectors in isolation to remodeling the TME itself to support sustained anti-tumor activity.

**Table 1:** Summary of TME remodeling strategies and their impact on ACT efficacy. Each approach is categorized by the primary TME barrier targeted, the mechanism by which remodeling occurs, representative preclinical or clinical evidence, and the observed effect on ACT outcomes.

Treatment strategy	Main TME barrier addressed	Mechanism of remodeling	Key findings	Impact on ACT
Vasculature reprogramming (anti-VEGF)	Abnormal, leaky, hypoxic tumor vasculature blocking immune infiltration (26)	Vessel normalization reduces leakiness, improves perfusion, oxygenation, and trafficking (27, 28)	VEGFR inhibitors transiently normalize vessels and improve radiotherapy/chemotherapy; “window of normalization” shown in glioblastoma trial (29, 30)	Enhanced T cell trafficking and delivery of ACT during normalization window
TAM remodeling with bacterial antigens (E. coli MG1655)	Immunosuppressive M2-like macrophages, chemokine barriers (31)	Bacterial colonization of hypoxic regions → TLR4/NF-κB activation → TAM shift to M1, CCL5 secretion (32)	In B16F10 melanoma, intratumoral MG1655 reprogrammed TAMs, boosted CD8^+^ infiltration, synergized with OT-I T cells (33)	Increased infiltration, cytokine production, tumor regression, prolonged survival with ACT
Oncolytic viruses (OVs)	Poor antigen presentation, TIL exhaustion (34–39)	OVs lyse tumor cells, release TAAs/DAMPs, can be armed with cytokines (IL-12, GM-CSF) or co-stimulatory ligands (OX40L)	Vaccinia virus OX40L/IL-12 converted tumor cells to “artificial APCs”; T-VEC FDA-approved; VSV-IFNβ study showed timing critical (34)	Promote TIL activation, epitope spreading, macrophage repolarization; but type I IFN (e.g. IFN-β) may impair CAR-T persistence if not timed properly
Cryo-thermal therapy (CTT)	Immunologically “cold” tumors lacking inflammation (40)	Freeze–heat cycles induce necrosis, DAMP release, sterile inflammation (41, 42)	In B16F10 and 4T1 models, CTT + ACT reduced metastases, reshaped systemic immunity, improved survival (~90%) (41, 42)	Synergistic systemic immune response, macrophage repolarization, distant tumor control
ECM stiffness modulation (FAK/LOX inhibition, CAF targeting)	Dense fibrotic ECM excludes T cells (43)	Inhibit FAK, integrin β1, or LOX → reduced collagen crosslinking, softer ECM, decreased YAP/TAZ signaling (44–51)	Preclinical models: improved infiltration and immune activation; early clinical trial (defactinib + ICI + chemo) showed ↑ cytotoxic T cells in biopsies (50)	Relieves physical and signaling barriers, enhances ACT infiltration.
Cytokine modulation (IFN-γ, IL-2)	Dysregulated cytokine networks limiting T cell survival and function (52–55)	Augment stimulatory cytokines (IL-2/IFNs) (53, 54)	ACT can be paired with cytokine modulation strategies to improve therapeutic efficacy and to sensitize tumors to ACT-mediated killing (55)	Support of the proliferation, activation, and survival of infused T cells. Acts as chemoattractant facilitating T cell trafficking.

VEGF, vascular endothelial growth factor; TKI, tyrosine kinase inhibitor; TAM, tumor-associated macrophage; TAA, tumor-associated antigen; DAMP, damage-associated molecular pattern; OV, oncolytic virus; T-VEC, talimogene laherparepvec; CTT, cryo-thermal therapy; ECM, extracellular matrix; FAK, focal adhesion kinase; CAF, cancer-associated fibroblast; LOX, lysyl oxidase.

As we look toward the future, even deeper questions emerge: what combination of interventions will work best, and in whom? Rather than searching for a single magic solution, success will likely require multi-pronged, mechanistically and individually informed strategies. To identify and optimize these, we need better experimental tools. Most current models fall short of capturing the spatial, mechanical, and cellular complexity of human tumors. This is where microphysiological systems offer a powerful advantage. By incorporating elements such as ECM stiffness, cellular heterogeneity, and tumor-associated microbiota, alongside continuous flow that mimics drug and cell delivery dynamics, they provide a more faithful *in vitro* representation of the TME. These platforms could significantly improve our ability to predict which therapeutic combinations will translate into clinical benefit.

Now, we need to consider that even with better models, one of the most urgent needs will be patient stratification. If combination therapies are to succeed, we must learn to match patients with the strategies most likely to benefit them. Can we define robust biomarkers of response or resistance? Could artificial intelligence (AI) uncover patterns that elude conventional analysis, helping clinicians design personalized ACT regimens based on tumor profiles?

Ultimately, progress in ACT will not come from a single breakthrough, but from the convergence of disciplines, technologies, and insights. Future success will depend on a deeply collaborative approach, uniting immunologists, bioengineers, data scientists, and clinicians. We also need to train and empower a new generation of researchers with transversal background, to bridge the gap between discovery and application. To truly unlock the full potential of ACT in solid tumors, we must stop treating immune cells and tumors as isolated actors and start modulating the entire ecosystem they inhabit.
